# Development and feasibility study of a piezoresistive pressure sensor-based automated system for monitoring and controlling gastric pressure in endoscopy

**DOI:** 10.1007/s11517-024-03254-1

**Published:** 2024-12-03

**Authors:** Sukgyu Koh, Sungwan Kim

**Affiliations:** 1 Interdisciplinary Program in Bioengineering, Graduate School, Seoul National University, Seoul, South Korea; 2 MedInTech Inc., 60, Daehak-Ro, Jongno-Gu, Seoul, South Korea; 3 Department of Biomedical Engineering, Seoul National University College of Medicine, Seoul, South Korea

**Keywords:** Endoscopes, Gastrointestinal, Equipment Design, Gastroenterology, Biomedical Engineering, Endoscopy, Gastrointestinal

## Abstract

Maintaining precise intragastric pressure during gastrointestinal endoscopy is critical for patient safety and diagnostic accuracy, yet current methods relying on manual adjustments pose risks of improper insufflation. This study aimed to develop an automated gastric pressure control system for flexible endoscopy, addressing these challenges with a piezoresistive pressure sensor that can be integrated into a 7.3 mm diameter flexible endoscope. The system, incorporating air and suction pumps controlled by a microcontroller, was calibrated in an acrylic chamber and validated through comprehensive testing in both an endoscopy simulator and a porcine specimen. Testing scenarios included normal breathing, coughing, belching, and combined events, assessing accuracy, stability, and real-time pressure regulation under conditions mimicking physiological responses. Results demonstrated high accuracy (R^2^ = 0.9999), minimal bias (0.23 mmHg), and strong agreement with reference standards, confirming effective pressure management. Simulated clinical scenarios in simulator and porcine specimen further showed the system’s ability to maintain target pressure with minimal errors, indicating robustness under dynamic conditions. These findings suggest that the automated pressure control system significantly improves safety and procedural efficiency in endoscopy, with potential applicability to other minimally invasive procedures. Further animal model testing is recommended to validate the clinical performance under realistic physiological conditions.

**Graphical Abstract:**

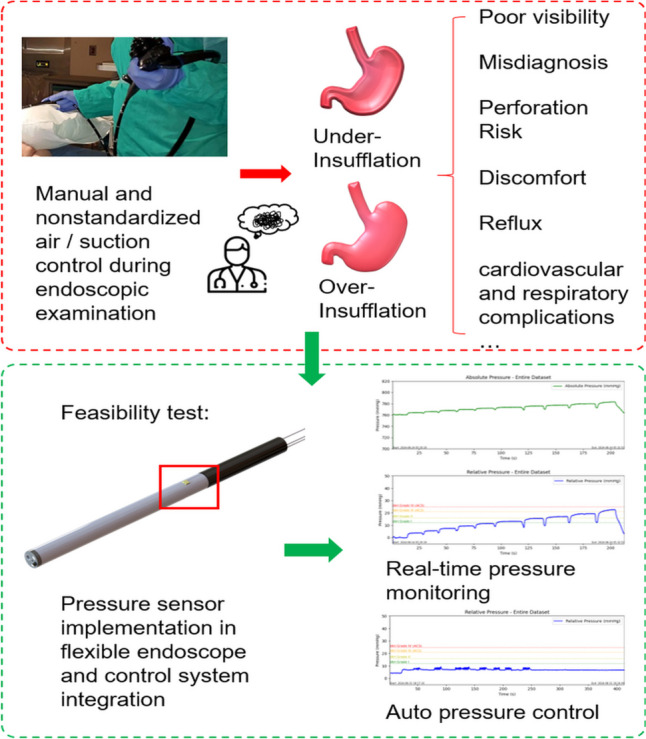

## Introduction

Endoscopy has become essential in the field of gastroenterology because of its real-time imaging capabilities and noninvasive nature, benefiting both human and veterinary medicine [1]. Endoscopy systems allow medical professionals to observe and diagnose conditions within the gastrointestinal (GI) tract (the system of organs, including the stomach and intestines) without the need for major surgery, thereby significantly reducing the risk and cost of sedative agents, recovery time, and minimizing stress on patients [2]. Additionally, endoscopy is highly repeatable, allowing for ongoing monitoring of disease progression or treatment outcomes, making it particularly useful in longitudinal studies.

A critical aspect of flexible GI endoscopy is the management of intragastric pressure through endoscopic insufflation, which directly impacts the quality of visualization and procedural safety. Insufflation, typically achieved by introducing air or carbon dioxide into the GI tract, creates adequate distension of the lumen, allowing for enhanced visibility during endoscopic examination [3]. However, precise control of the insufflation pressure is necessary to prevent complications arising from over- and under-insufflation. Currently, pressure is manually adjusted during procedures without standardization, leading to user deviations and issues with both under- and overinsufflation, potentially compromising the safety and accuracy of the diagnosis [4]. Inadequate insufflation can lead to diagnostic errors, potentially missing early cancer indicators due to poor visualization, which is crucial for patient survival [5]. On the other hand, while serious complications during upper gastrointestinal endoscopy are rare, gastric over-distention from excessive insufflation is the most common complication. Overinsufflation can be life-threatening, as excessive gastric distension can result in reduced venous return, lower blood pressure, and decreased tidal volume [6] and can cause severe adverse events such as Mallory‒Weiss syndrome (tears in the esophagus) [7], Boerhaave syndrome (rupture in the esophagus) [8], or acute peritonitis (rupture in the stomach) [9].

Patient comfort is also a significant consideration during endoscopy. In human procedures, sedation is commonly used to increase comfort and satisfaction, with sedatives such as midazolam or propofol being frequently administered [10]. In contrast, veterinary endoscopy typically requires general anesthesia [11]. In human patients, when sedation is used instead of general anesthesia, gastric overdistension can often be mitigated by encouraging belching through communication. However, this approach is not feasible in anesthetized animals, making close monitoring of insufflation crucial. Uncontrolled air introduction can lead to overinsufflation, resulting in cardiovascular and respiratory complications [11]. If gastric overinflation is detected in anesthetized patients, the stomach can be deflated by using the endoscope to suction out air, which is why a suction pump is a critical component of endoscopy equipment [6, 12]. If there is no suction pump, manually pressing on the abdomen can sometimes reduce stomach distension by causing forced burping around the endoscope. If this does not work, a tube may need to be inserted through the mouth into the stomach to relieve bloating [6].

Various devices that automatically regulate pressure, such as Pneumo Sure™ High Flow Insufflator (Stryker, Selzach, Switzerland), UHI-4 insufflator (Olympus, Tokyo, Japan), and GW-200 (Fujifilm, Tokyo, Japan), have been developed and are commercially available for use in laparoscopy. However, automatic insufflation with pressure control has not yet been practically implemented in flexible GI endoscopy, primarily due to a lack of suitable equipment [13]. Recently, a system has been developed to attach to commercially available automatic pressure regulators for laparoscopy, enabling automatic pressure control during diagnostic flexible endoscopy [14]. Despite its innovation, this device has limitations, including the need for a commercial overtube with a 19.5 mm diameter that attaches to the endoscope's insertion tube, potentially increasing patient discomfort during the examination. Additionally, the longer overtube required for maintaining consistent pressure beyond the esophagus and the additional leak-proof device can both hinder the handling of the flexible endoscope. Moreover, current surgical insufflators are designed primarily for intraperitoneal use rather than for GI applications, leading to an imbalance between insufflation and evacuation [14].

To address these limitations, there is a significant need for endoscopic devices with integrated, real-time pressure monitoring and automated control systems tailored specifically for GI endoscopy. However, to the best of our knowledge, no current flexible endoscopes on the market offer real-time pressure measurement or control systems specifically designed for gastrointestinal procedures. This study aims to address this gap by evaluating the feasibility of a piezoresistive MEMS pressure sensor for real-time monitoring and control of gastric pressure. The goal is to develop an automated gastric pressure control system that can be directly integrated into flexible endoscopy systems to increase patient safety, improve procedural efficiency, and mitigate risks associated with improper insufflation, particularly in anesthetized patients.

## Methods

### Sensor design

**Fig. 1:**
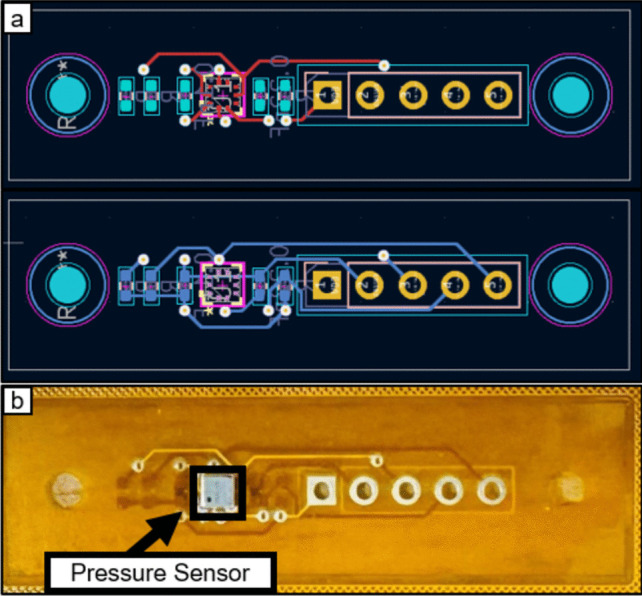
(a) Front and back layout of the 2-layer flexible printed circuit board (FPCB) design and (b) fully fabricated sensor with the pressure sensor in position

The sensor utilized in this system was chosen after careful and thorough consideration of its performance characteristics and environmental suitability. Owing to the challenging conditions within the GI tract, it is crucial to choose a sensor that offers consistent accuracy and can withstand exposure to bodily fluids. The design incorporates a waterproof piezoresistive pressure sensor (BM1390GLV, ROHM, Japan), with dimensions of just 2 mm × 2 mm × 1 mm, mounted on a flexible printed circuit board (FPCB) measuring 10 mm × 37 mm × 0.11 mm, as illustrated in Fig. 1. This compact design effectively minimizes space usage, making it ideal for integration into small diagnostic devices. For flexible endoscopy applications, the sensor is connected with approximately 3 m of 32 AWG wire for data communication. This length is sufficient to position the sensor at the tip of the insertion tube, ensuring reliable data transmission to the processing microcontroller located at a distance.

### Overall control and monitoring system

**Fig. 2:**
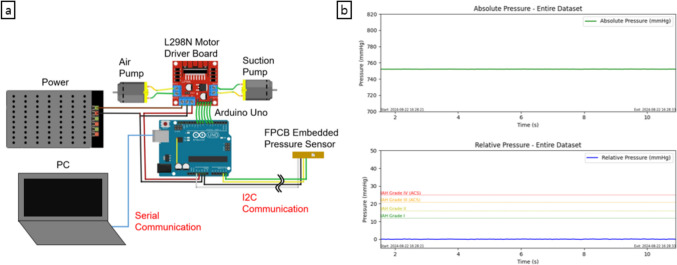
(a) Schematic overview of the automated gastric pressure control system. (b) Real-time monitoring output, with the first plot showing the absolute pressure and the second plot showing the relative pressure

To achieve automatic pressure control, as shown in Fig. 2a, two air pumps (D61-BA301, BHL Co. Ltd., South Korea) were connected to an L298N motor driver board, which interfaced with a microcontroller (Arduino Uno, Arduino, Italy). The air pumps used for insufflation control are equipped with a 12 V DC motor rated at 7.8 Watts, providing reliable performance for sustained operations. At its rated load, the motor operates at 1650 RPM, ensuring efficient airflow for pressure regulation. The pump is capable of delivering a free flow rate of 8 L/min, making it suitable for applications requiring controlled air supply and suction. The system uses the air pump outlet for the air function and the inlet for the suction function of each pump. Sensor data were transmitted to the microcontroller via I2C communication through approximately 3 m of wire for real-time monitoring and data logging. The data were then displayed in real-time plots on a computer through serial communication.

The plot window of the monitoring interface was arranged into a 1 × 2 grid of subplots: the first plot displayed cumulative absolute pressure data in mmHg from the start of recording, whereas the second plot showed relative pressure with respect to atmospheric pressure, as illustrated in Fig. 2b. Although the default zero reference is set to atmospheric pressure, the user can reset the zero reference point at any desired location, such as the symphysis pubis bone (joint where the two halves of the pelvis meet in front, located just above the genitals) [15] or the midaxillary line (imaginary line that runs vertically down the side of the body, starting from the middle of the armpit) [16]. Additionally, the user can toggle automatic air and/or suction control during operation. The relative pressure plot included markers for intra-abdominal hypertension at 12, 16, 21, and 25 mmHg, following the definitions established by the World Society of the Abdominal Compartment Syndrome (WSACS) [17]. This feature provides additional context, particularly for potential future applications of sensor technology in clinical settings where intra-abdominal pressure (IAP) monitoring is crucial. Data for both absolute and relative pressures were logged, allowing for detailed postexperiment analysis. Furthermore, the final plot image was automatically saved when the program was closed, including the starting and ending times for reference.

### Endoscope integration

**Fig. 3:**
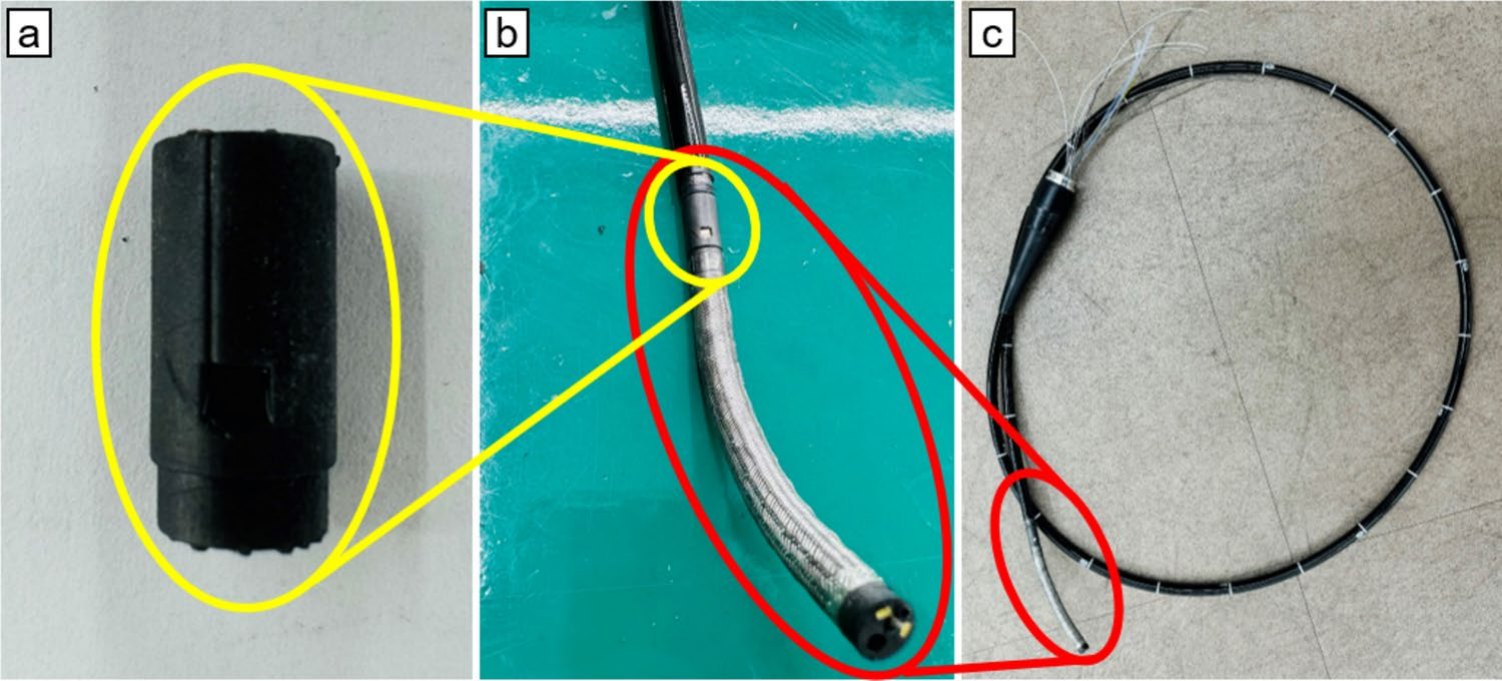
(a) A sensor mount link was designed and 3D printed for checking integration. (b) The sensor mount is positioned between the end of the insertion tube and the beginning of the bending section. (c) Assembled insertion part for a veterinary endoscope with a 7.3 mm outer diameter, successfully integrating the sensor along with LED wires, bending wires, a suction hose, an air/water hose, and image sensor cables within the tube

After calibration and testing, the sensor was embedded within a mechanically designed part created via 3D CAD and positioned between the endoscope’s insertion tube and the bending section. The sensor position was chosen to minimize direct contact with liquids and the GI wall and protect the sensor from the impact of air insufflation through the air hole at the distal tip, as shown in Fig. 3. Unlike a regular PCB, which cannot be easily embedded in such confined spaces, a flexible PCB was used to allow integration into the endoscope's compact structure, accommodating all necessary components, such as the image sensor, LED light source, air‒water hose, suction hose, and mechanical cables. Although all the components were included in the construction process, basic endoscopic functions, such as manipulation of the bending section, imaging, and lighting, were not implemented in this study. The primary focus was to validate the integration of the sensor into the compact design of the endoscopy system with an outer diameter as small as 7.3 mm and to test the feasibility of the automatic gastric pressure control system rather than developing a complete endoscopy system.

The operation of both pumps in this study was controlled by an adaptive proportional-integral-derivative (PID) mechanism to maintain the system pressure at a target value of 10.0 mmHg, which was based on the median pressure inside the digestive tract observed in a previous study during endoscopic diagnosis [18]. The adaptive PID controller, which calculates an error value as the difference between the desired setpoint and the actual measured pressure, was chosen for its capacity to provide stable and responsive pressure adjustments. This approach enables precise pressure control via real-time sensor readings, with accuracy down to two decimal places. To optimize the pressure control system's performance, the control parameters—proportional (P), integral (I), and derivative (D) gains—were set manually. The control strategy included both aggressive and conservative tuning parameters to optimize the system's response to varying pressure deviations. Specifically, when the pressure deviation from the target was substantial, the controller switched to aggressive parameters— aggressive P: 40, aggressive I: 10.05, and aggressive D: 20.0—to enable a faster response. When the pressure deviation was minimal, the controller adopted conservative settings— conservative P: 4, conservative I: 10.2, and conservative D: 40—to maintain stability and avoid overshoot. This adaptive approach allowed precise pressure control through real-time sensor readings, achieving accuracy down to two decimal places. The controller continuously monitored the pressure readings and dynamically adjusted the pump speeds to maintain the desired pressure level. When the system reached 10.0 mmHg, the pump automatically stopped to prevent unnecessary operation and reduce equipment wear. In the case of any deviation from the target, the adaptive PID algorithm finely tuned the pump speed adjustments, offering a more precise and reliable response than conventional PID control, which enhances both the safety and efficacy of the procedure.

### Experimental protocol

#### Acrylic chamber calibration

**Fig. 4:**
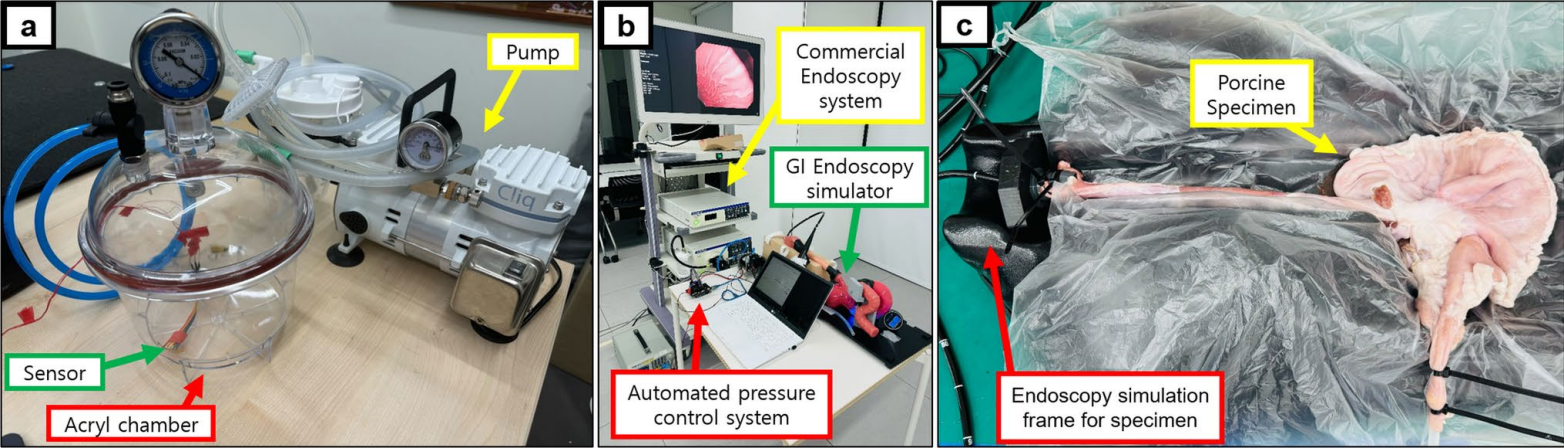
(a) Sensor calibration setup with a pressure sensor inside an acrylic chamber equipped with a pressure gauge and pump, (b) test environment with an automated pressure control system connected to a commercial endoscope, inserted and sealed in a human-like silicone upper GI endoscopy simulator, and (c) porcine esophagus-stomach-duodenum specimen

To evaluate the feasibility and calibration of the developed sensor module, an acrylic chamber with airtight seals was used to create a controlled environment where the internal pressure could be manipulated via an external pump, as shown in Fig. 4a. This setup provided a stable, controllable environment to establish baseline sensor accuracy under ideal conditions. The pressure sensor module was inserted into the chamber, with internal pressure regulated by gauge values, which served as the gold standard for calibrating the sensor readings. As the pump operated and the gauge values stabilized, a 3-s period was allowed for the sensor readings to stabilize. Subsequently, 20 data points immediately before each increment were collected and averaged for analysis. The pressure was gradually increased from −100 mmHg to −675 mmHg at 25 mmHg intervals. Using the relationship between the sensor readings and the gauge values, the sensor was calibrated and tested again via the same process. This iterative procedure was designed to assess the sensor module's accuracy across a range of pressures thoroughly.

Additionally, the sensor module underwent a 24-h continuous monitoring test in a laboratory setting to evaluate its durability and reliability, simulating extended use under clinical conditions.

#### Upper GI simulator test

Following successful calibration and durability testing in the acrylic chamber, the sensor was connected to the automated pressure control system and further evaluated in a more human-like environment via an upper GI simulator (LM-103, Koken Co. Ltd., Japan), as shown in Fig. 4b. This simulator replicates realistic conditions to assess the integration of the automated gastric pressure control system with an endoscopic setup. The GI simulator is assumed to provide a sufficiently realistic environment for testing the system’s ability to regulate pressure in clinical conditions. This implies that the simulator's physical properties, such as pressure and fluid dynamics, closely resemble those of the real gastrointestinal tract. The Olympus EVIS LUCERA ELITE 290 series (CV-290, CLV-290, GIF-H290, Olympus, Japan) was used for endoscopic simulation, with the sensor module positioned at the beginning of the endoscope's bending section. Securing all connections between simulator components prevents pressure loss, whereas sealing the esophagus portion after endoscope insertion mimics the tonic contraction of the lower esophageal sphincter [19]. Air and suction pumps, connected via a Y splitter to the suction nozzle of the GIF-H290, enabled automated gastric pressure control on the basis of real-time sensor readings within the simulated abdomen. This setup allowed for a comprehensive assessment of the functionality of the automatic pressure control system.

**Fig. 5:**
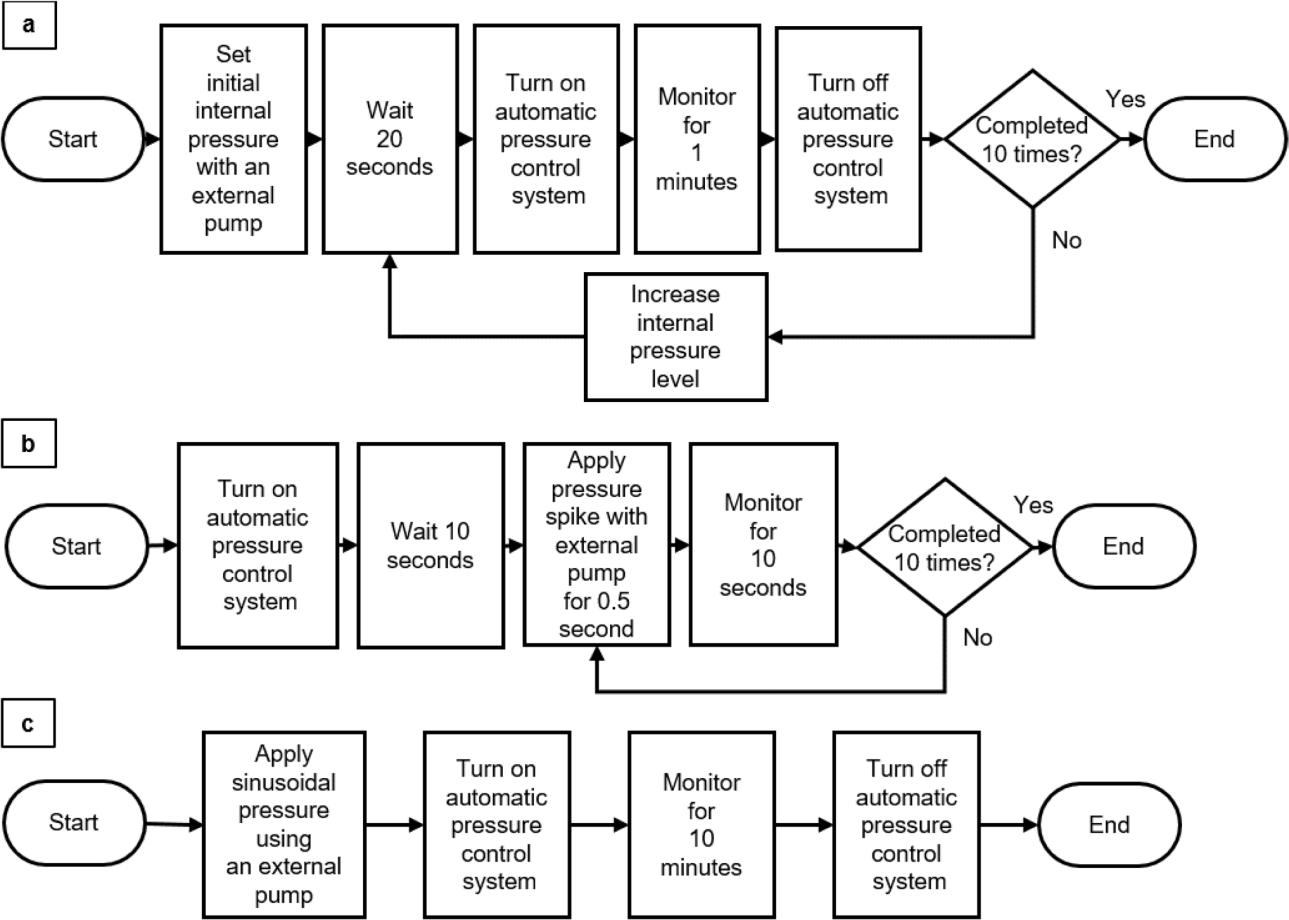
Flowchart of test procedures for (a) response time and stability tests at different initial pressure values, (b) stability tests during sudden pressure spikes simulating coughing and belching, and (c) stability tests during continuous pressure fluctuations simulating respiration

The test procedure aimed to evaluate the system's dynamic response in maintaining gastric pressure within the optimal range under varying conditions. The pressure inside the stomach model was incrementally increased in steps of 10 using a separate pump. At each step, the system allowed 20 s for the pressure to stabilize before the automatic gastric pressure control system was activated to determine if it would automatically adjust to regulate the gastric pressure to the desired level. The system was then monitored until the elapsed time reached 1 min. The automatic gastric pressure control system was turned off between each cycle to set different initial pressures. The response time from the initial pressure to the steady-state pressure was recorded, with the timer stopping when the moving average of 5 data points reached 10 mmHg ± 0.5 mmHg. The mean value, standard deviation, and 95% confidence intervals at the steady-state pressure were calculated and recorded to analyze the system's consistency and reliability over the 1-min operation. The experimental procedure is shown in Fig. 5a.

To evaluate the system's performance in handling realistic physiological fluctuations, we introduced additional test conditions to simulate coughing, belching, normal breathing, and random combinations of these events. These tests were designed to assess the system's ability to maintain pressure control under dynamic conditions.

**Coughing simulation:** After reaching a steady state by activating the pressure control system, we simulated abrupt pressure spikes to mimic coughing as described in Fig. 5b. This was achieved by applying the maximum external air pump speed 10 times, with each spike testing the system's ability to compensate for sudden pressure fluctuations and return to steady-state conditions. On the basis of a reported study, the abdominal pressure during coughing reaches levels too high (approximately 157.7 mmHg for males and 121.4 mmHg for females) to be replicated in our experimental setup [20]. Therefore, the maximum pump speed was used to create sudden upward spikes at 0.5-s intervals. The pressure was monitored for 10 s before initiating the next spike. Although the amplitude was smaller than the reported physiological pressures, this test allowed us to observe the system's reliability with respect to sudden spikes.

**Belching simulation:** The same procedure was used to simulate belching, but this time, the external pump’s air inlet was connected to create negative pressure spikes. Since we could not find specific reports on pressure reduction during belching, the maximum pump speed was used again to create a significant pressure drop, simulating an extreme scenario.

**Normal breathing simulation:** Following the pressure spike tests, we simulated continuous pressure fluctuations to represent normal respiratory movements. The experimental procedure is depicted in Fig. 5c. This test was conducted for 10 min to evaluate the system's stability during regular breathing cycles. The pressure amplitude (± 2 mmHg) [21] and breathing frequency (mean 16 cycles per minute) inside the stomach were referenced from a previously reported study [22].

**Random coughing and belching events during normal breathing:** Finally, we evaluated the system’s ability to regulate pressure during randomly occurring coughing and belching events within a normal breathing pattern. The same procedure for normal breathing shown in Fig. 5c was used; however, ten coughs and ten belches were randomly distributed throughout a 10-min experiment, and the system's performance in maintaining steady pressure despite these random fluctuations was assessed.

The analysis of pressure data for each experimental mode (coughing, belching, breathing, and random events) involved calculating the mean and standard deviation of pressure, the mean and standard deviation of response time, and the steady-state error to evaluate the system's performance under different conditions. Data were collected continuously and recorded in text files containing columns for elapsed time, relative pressure (mmHg), and external motor speed. The analysis began by filtering the data to isolate relevant segments starting from the point where significant changes in pressure occurred. For the coughing and belching modes, this point was determined as the first instance where the external motor speed exceeded zero. For the breathing and random event modes, the analysis began when the relative pressure first reached or exceeded the target pressure of 10 mmHg. Pressure spikes were identified by detecting changes in the external motor speed that surpassed a defined threshold, which was set at 50 PWM for this study, while the first spike was excluded to avoid any initial instability affecting the results. The response time for each segment was calculated by determining the time taken for the pressure to reach and remain within ± 0.1 mmHg of the target pressure (10 mmHg). This response time was measured from the moment the spike began until the specified pressure range was achieved.

#### Porcine esophagus-stomach-duodenum specimen test

In addition to the GI simulator, the system's performance was evaluated using a porcine esophagus-stomach-duodenum specimen placed on a GI simulation model (EASIE-R4 Simulator, EndoSim, LLC, USA). This specimen provided a more physiologically accurate environment to assess the system's functionality under conditions closely resembling a real digestive tract. The pressure sensor and air/suction hose were fixed at the end of the endoscope's insertion tube using a glue gun to ensure secure attachment and waterproofing. The endoscope was then inserted into the stomach through the esophagus. To minimize air leakage, both the esophagus and duodenum were sealed via cable ties, providing a more controlled and airtight environment for the experiment to ensure more accurate simulation of real physiological conditions. The same experimental procedures described in Sect. 2.4.2 were conducted on the specimen to test the system's response to simulated physiological fluctuations, including coughing, belching, breathing, and mixed scenarios. These additional tests helped validate the system's ability to maintain stable pressure control under more realistic clinical conditions, providing a comprehensive evaluation of its performance across different testing environments.

### Statistical analysis

The R^2^ correlation between the sensor module's readings and the reference pressure gauge was calculated to evaluate the strength of the linear relationship between the two measurement methods. Additionally, the pressure readings from the developed sensor module were compared with those from the reference pressure gauge via Bland‒Altman analysis to assess bias and precision [23]. The mean bias, along with the standard deviation (SD) and standard error (SE), was calculated. Moreover, the 95% confidence intervals (CI) for the lower and upper limits of agreement were determined. Furthermore, Bland‒Altman analysis was conducted with respect to the optimal pressure of 10 mmHg when the automated function was enabled in the GI simulator and porcine specimen. All plots were generated, and significant values were calculated and analyzed via a Python program.

## Results

### Sensor calibration and stability test

**Fig. 6:**
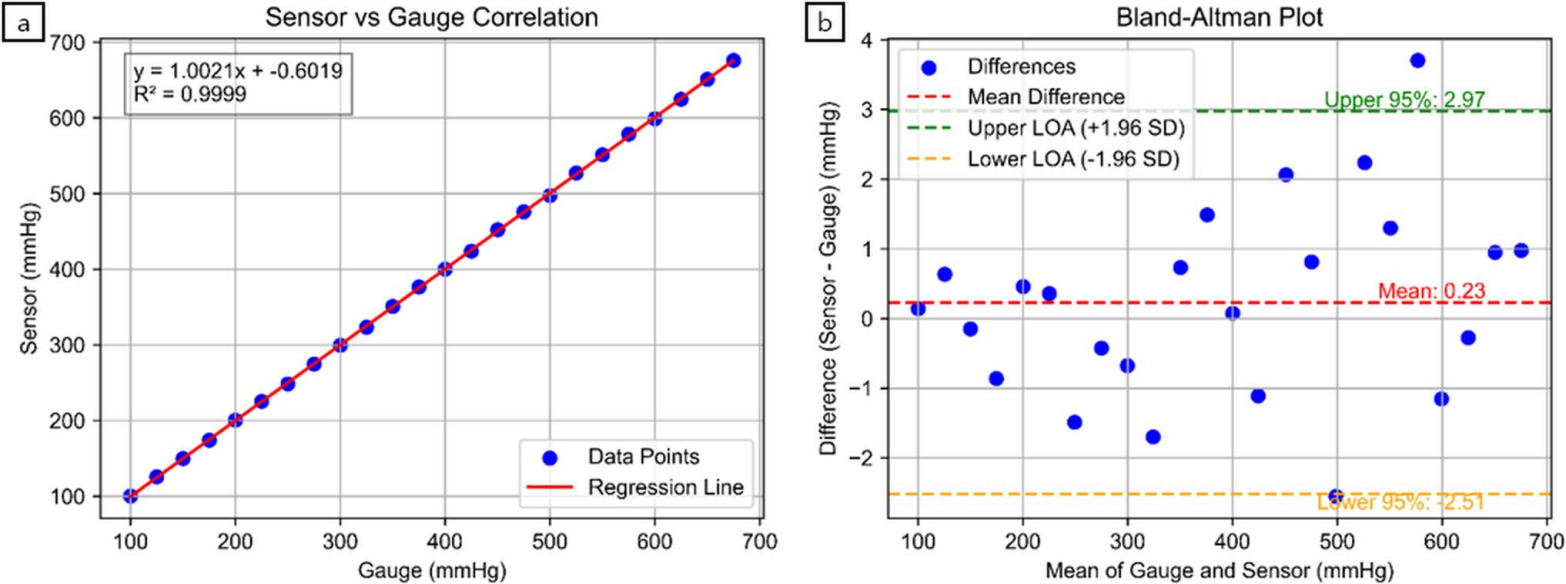
(a) Correlation plot between the sensor module and gauge. (b) Bland‒Altman analysis comparing the sensor module and gauge measurements

This study aimed to evaluate the feasibility and accuracy of a piezoresistive MEMS pressure sensor for monitoring and controlling gastric pressure. As part of this, we calibrated the sensor module to assess its reliability in providing real-time pressure measurements. The sensor was successfully calibrated within an acrylic chamber, demonstrating exceptionally high accuracy, with an R^2^ correlation of 0.9999 between the sensor readings and the reference pressure gauge, indicating an exceptionally strong linear relationship (Fig. 6a). This strong correlation supports the sensor’s ability to function reliably for clinical applications, particularly in the context of automated pressure control in endoscopy.

Bland‒Altman analysis (Fig. 6b) revealed a mean bias of 0.2308 mmHg, with a standard deviation of 1.4001 mmHg and a standard error of 0.2858 mmHg. The lower 95% confidence interval was −2.5133 mmHg, and the upper 95% confidence interval was 2.9750 mmHg, indicating minimal bias and high precision (Table 1). These results confirm that the sensor module provides accurate pressure measurements comparable to those of a reference gauge, with minimal bias and high precision.

**Table 1:** Bland‒Altman analysis for sensor calibration in the acrylic chamber and steady-state intervals during a 10-min operation in the upper GI endoscopy simulator and porcine specimen; all values are in mmHg (SD: standard deviation, SE: standard error, L95% CI: lower 95% confidence interval, U95% CI: upper 95% confidence interval)

	Mean difference	SD	SE	L95%CI	U95%CI
Calibration acrylic chamber	0.2308	1.4001	0.2858	−2.5133	2.9750
Upper GI endoscopy simulator	−0.4982	0.6789	0.0065	−1.8290	0.8325
Porcine specimen	0.6374	0.0667	0.0006	0.5066	0.7682

After the calibration process, a total of 1,808,087 data points were collected over a continuous 24-h period, as summarized in Table 2. The data were initially recorded over a period slightly exceeding 24 h. To ensure consistency and focus the analysis on a precise timeframe, the data were truncated at the 24-h mark, resulting in 1,665,281 data points used for subsequent analysis and reporting. The consistent timing of the data logging events, with a mean time difference of 0.0519 s and a standard deviation of 0.0026 s, demonstrates the system's reliability in terms of temporal resolution and accurate long-term pressure measurement. The mean relative pressure was −0.2862 mmHg, with minimal deviation from the baseline pressure, confirming the system's ability to maintain stable pressure over time.

**Table 2:** Overview of 24-h continuous pressure monitoring results (SD: standard deviation)

Total data count	Mean relative pressure (mmHg)	Relative pressure SD (mmHg)	Mean time difference (s)	Time difference SD (s)
1665281	−0.2862	0.8543	0.0519	0.0026

### Upper GI endoscopy simulator and porcine specimen test

#### 10-min stability test

**Fig. 7:**
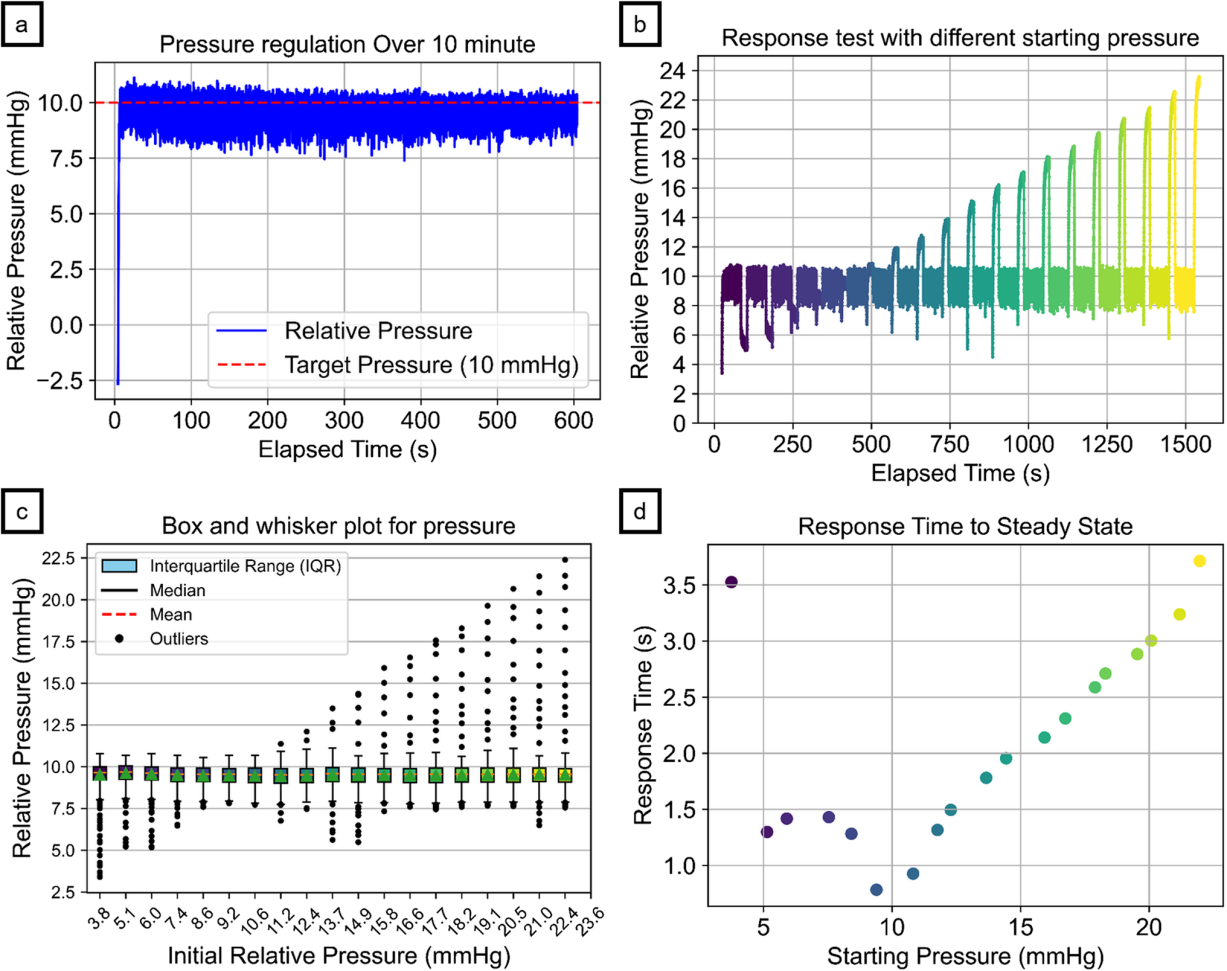
(**a**) Stability test for 10 minutes with the setpoint at 10 mmHg; (**b**) automated gastric pressure control system maintaining a setpoint at 10 mmHg across 19 incremental initial pressure steps; (**c**) box and whisker plot for each step’s steady-state period; (**d**) response time from initial pressure to steady-state pressure across 19 steps in the GI simulator

In line with our research question about whether the automated system could reliably maintain target pressure during endoscopy, the system was tested in both an upper GI simulator and a porcine esophagus-stomach-duodenum specimen. The results aligned with the calibration findings, further verifying the system’s clinical potential. The system effectively maintained the target pressure of 10 mmHg, as shown in Fig. 7a–c, demonstrating high reliability in pressure control. Bland‒Altman analysis values were calculated based on steady-state pressure and sensor readings. The mean difference between the sensor module readings and the target pressure was −0.4982 mmHg in the GI simulator and 0.6374 mmHg in the porcine specimen, with standard deviations of 0.6789 mmHg and 0.0667 mmHg, respectively, as shown in Table 1. These results indicate a high degree of agreement between the sensor module and the target pressure, supporting the system’s safe and consistent operation across both testing environments**.** The low standard errors for both the GI simulator (0.0065 mmHg) and the porcine specimen (0.0006 mmHg) suggest minimal variability in the pressure measurements. The narrow range of the 95% confidence interval, with a GI simulator from −1.8290 to 0.8325 mmHg and a porcine specimen from 0.5066 to 0.7682 mmHg, further underscores the system's precision and reliability, showing that pressure readings consistently aligned closely with the target level in both testing environments. After reaching the steady-state pressure, the air and suction pumps were alternately activated to maintain the internal pressure within an acceptable range. This process ensured continuous pressure control throughout the test duration, allowing the system to adjust effectively to any minor fluctuations in pressure.

The system response time also showed effective regulation, as shown in Fig. 7d, revealing a nonlinear relationship between the initial pressure and the time required to reach the steady-state pressure of 10 mmHg. The response time was longest (approximately 3.71 s) when the initial pressure was 23.6 mmHg, whereas at an initial pressure of 3.8 mmHg, the system took 3.53 s to stabilize. Although the target pressure was 10 mmHg, the mean steady-state pressure was observed near 10.36 mmHg, further confirming the system's ability to approach the desired pressure levels with high accuracy.

#### Coughing simulation

**Fig. 8:**
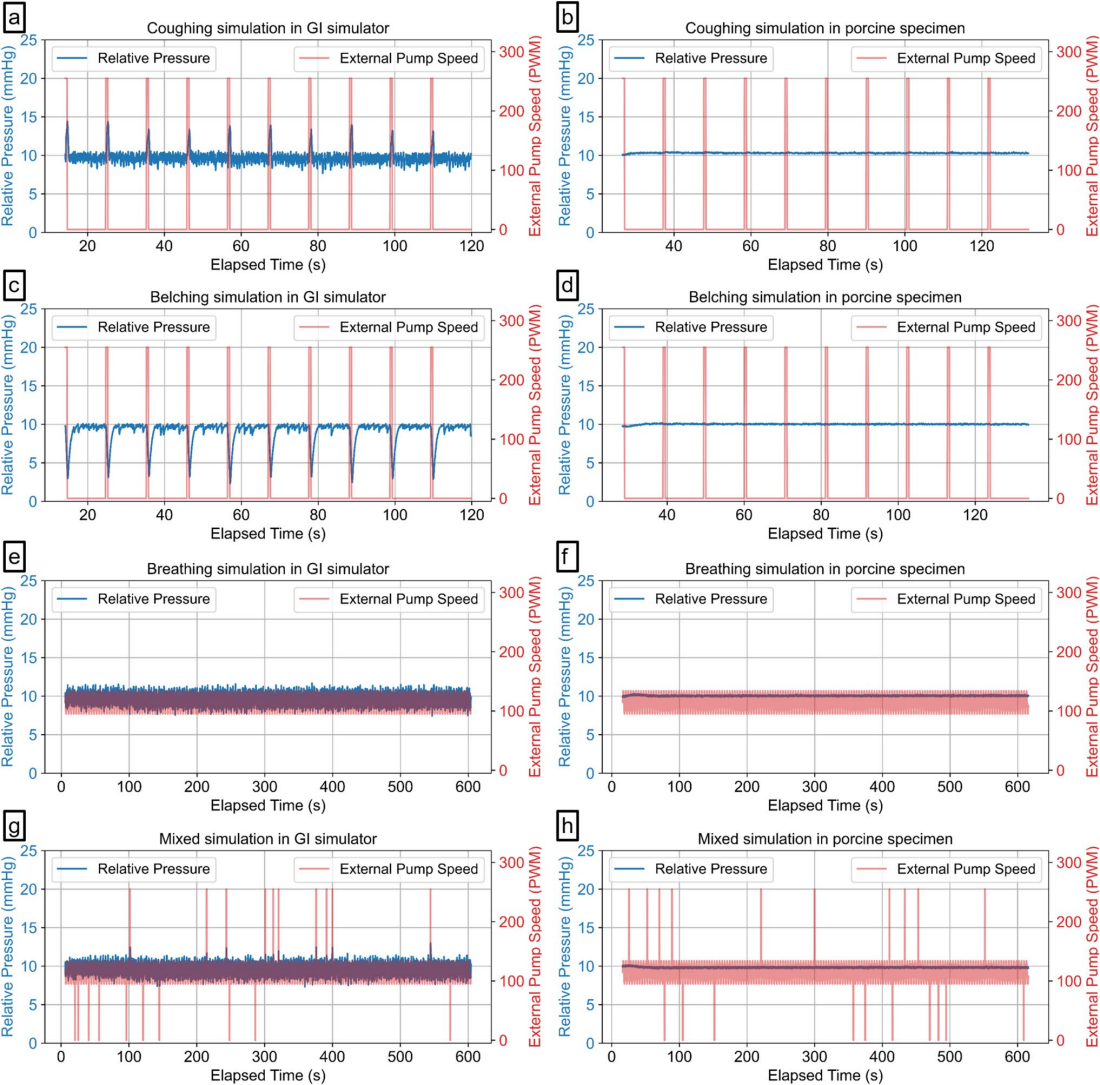
Automatic pressure control system results from simulations of (a) coughing in the GI simulator, (b) coughing in the porcine model, (c) belching in the GI simulator, (d) belching in the porcine specimen, (e) normal breathing in the GI simulator, (f) normal breathing in the porcine specimen, (g) combined events of coughing, belching, and breathing in the GI simulator, and (h) combined events in the porcine specimen

During simulated coughing, as shown in Fig. 8a and Table 3, the system maintained a mean pressure of 9.74 mmHg with a standard deviation of 0.91 mmHg in the GI simulator and a standard error of 0.0206 mmHg. The mean response time, defined as the time required to return to the target pressure following each abrupt pressure spike, was 0.83 s with a standard deviation of 0.44 s. In the porcine specimen, the mean pressure was slightly higher at 10.30 mmHg with a standard deviation of 0.06 mmHg and a standard error of 0.0013 mmHg. Response time metrics were not applicable since there were no noticeable fluctuations in the sensor readings following each spike, as described in Fig. 8b, possibly due to tissue elasticity.

**Table 3:** Statistical Data for Simulated Physiological Conditions (SD: standard deviation, SE: Standard Error, n/a: not applicable)

	Model	Mean Pressure (mmHg)	Pressure SD (mmHg)	SE (mmHg)	Response Time (s)	Response Time SD (s)
Coughing	GI Simulator	9.7394	0.9080	0.0206	0.8267	0.4371
	Porcine Model	10.3039	0.0588	0.0013	n/a	n/a
Belching	GI Simulator	9.1007	1.4727	0.0334	2.2547	1.2189
	Porcine Model	10.0155	0.0678	0.0015	n/a	n/a
Normal breathing	GI Simulator	9.6400	0.6772	0.0065	n/a	n/a
	Porcine Model	10.0488	0.0550	0.0005	n/a	n/a
Random coughing and belching during normal breathing	GI Simulator	9.6273	0.7068	0.0068	0.5862	0.4783
	Porcine Model	9.8280	0.0735	0.0007	n/a	n/a

#### Belching simulation

For the belching simulations, as illustrated in Fig. 8c, the system achieved a mean pressure of 9.10 mmHg with a standard deviation of 1.47 mmHg and a standard error of 0.0334 mmHg in the GI simulator. The mean response time was 2.25 s with a standard deviation of 1.22 s, reflecting the system's ability to recover from more significant negative pressure drops introduced during belching. For the porcine specimen, the system maintained a mean pressure of 10.02 mmHg with a standard deviation of 0.07 mmHg and a standard error of 0.0015 mmHg. The response time metrics were again marked as not applicable, as there were no noticeable fluctuations in the sensor readings, indicating a sharp recovery period, as depicted in Fig. 8d.

#### Normal breathing simulation

During the normal breathing simulation, as illustrated in Fig. 8e, the system maintained a stable mean pressure of 9.64 mmHg with a standard deviation of 0.68 mmHg and a standard error of 0.0065 mmHg in the GI simulator. For the porcine specimen, the mean pressure remained similarly stable at 10.05 mmHg, with a standard deviation of 0.05 mmHg and a minimal standard error of 0.0005 mmHg, as shown in Fig. 8f. Again, response time metrics were not applicable for either test.

#### Random coughing and belching events during normal breathing

In the scenario combining normal breathing with abrupt coughing and belching spikes, as shown in Fig. 8g, the system maintained a mean pressure of 9.63 mmHg with a standard deviation of 0.71 mmHg and a standard error of 0.0068 mmHg in the GI simulator. The mean response time was 0.59 s, with a standard deviation of 0.48 s, demonstrating the system’s responsiveness to complex pressure fluctuations. For the porcine specimen, as depicted in Fig. 8h, the system maintained a mean pressure of 9.83 mmHg with a standard deviation of 0.07 mmHg and a standard error of 0.0007 mmHg. Similarly, the response time metrics for the porcine specimen were not applicable because of the absence of noticeable fluctuations in the sensor readings.

## Discussion

The results of this study demonstrate that the sensor module can be effectively integrated into the compact structure of a flexible endoscope, providing stable and highly accurate measurements for intragastric pressure sensing and monitoring. This is evidenced by a strong correlation with a reference pressure gauge, indicating high measurement accuracy and near-perfect linearity. The Bland‒Altman analysis further supported this finding, with a small mean bias and a narrow 95% confidence interval, confirming minimal variability and high agreement (Table 1, Fig. 6a, 6b). These findings confirm that the sensor module can provide accurate and stable pressure measurements comparable to a reference standard, making it suitable for clinical applications. The waterproof design of the sensor enhances its durability, ensuring that it maintains its accuracy and performance even in challenging environments, making it ideal for continuous monitoring during endoscopic procedures. The results of this study suggest that incorporating MEMS technology into flexible endoscopes can significantly increase the precision of intragastric pressure management.

During the 24-h continuous monitoring period, the system demonstrated a slight negative bias from the baseline pressure, possibly due to calibration drift over time, influenced by environmental factors such as temperature fluctuations. Addressing these potential influences through temperature-compensated sensor designs could further improve long-term accuracy. The consistent time intervals between consecutive data logging events indicate reliable data capture at a steady rate, ensuring near real-time monitoring. This capability is crucial for accurately detecting and recording rapid pressure changes, which is essential for the timely diagnosis and management of medical conditions. Overall, these results confirm the system’s capability for reliable and consistent pressure monitoring, with the potential for further refinement to improve accuracy and adaptability in various clinical settings.

The GI endoscopy simulator tests reinforced the sensor's calibration, showing effective maintenance of the target pressure at 10 mmHg with a high level of agreement and a narrow range of 95% confidence intervals. The system’s ability to quickly respond to pressure deviations, particularly at different initial pressures, underscores its effectiveness in dynamic clinical environments. For the response test, the time required to increase pressure was longer than the time required to decrease it; however, minimizing the time to reduce pressure when overinflated is more critical, as it directly affects the risk of potential complications. The nonlinear trend and extended steady-state response times may result from inadequate PID tuning and limitations in the motor driver and pump performance. Constraints such as the capped motor speed and low speed resolution may have contributed to the system's response delays. The adaptive parameters might be specifically tuned with gain settings that prioritize rapid stabilization in the 4–8 mmHg range. With enhanced components and more precise tuning, the system's response time could be significantly improved, potentially achieving faster and more efficient adjustments.

Simulated physiological events, including coughing, belching, and normal breathing, offered further insights into system robustness. During the coughing simulation, the system managed to maintain pressure control with a mean pressure close to the target, despite the variability introduced by sudden pressure spikes. The short response time and low standard error suggest that the system can quickly adjust to rapid upward pressure changes, making it suitable for scenarios where abrupt fluctuations are common. These results are encouraging for potential clinical scenarios, where similar sudden pressure changes could occur during a procedure. The belching simulation introduced negative pressure spikes, and although the system performed well, it presented slightly higher standard errors than did the coughing simulation. This suggests that while the system is well tuned for handling rapid increases in pressure, further adjustments may be needed to optimize the performance for sudden pressure drops. Nonetheless, the system's response times remained within clinically acceptable limits. During the normal breathing simulation, the system maintained stable pressure fluctuations consistent with physiological respiratory patterns, showing excellent stability and low variation in pressure measurements. This is particularly important in endoscopic procedures where continuous moderate pressure changes, such as those caused by breathing, need to be accurately managed to prevent over- or under-insufflation. The system’s ability to handle these subtle, continuous fluctuations with minimal error highlights its potential for routine clinical use, where precision is critical. The most complex scenario involving random coughing and belching events during normal breathing demonstrated the system's ability to maintain pressure control even under highly dynamic and unpredictable conditions. The results demonstrated that the system could maintain pressure control even under irregular and sudden fluctuations, with reasonable response times and low standard errors. These results show the system's robustness in handling a variety of physiological events, reinforcing its suitability for real-world clinical environments where such events are common.

In-depth analysis of the porcine specimen results reveals critical insights that extend beyond those derived from the GI simulator alone. Unlike the silicone material of the GI simulator, which has limited elasticity and results in immediate pressure fluctuations, the porcine tissue stretches more gradually, allowing for a smoother adaptation to changes in pressure. This balloon-like response of living tissue likely minimized sharp fluctuations, enabling the control system to maintain stable sensor readings without the abrupt response times required in the simulator environment. The consistent and stable results observed in the porcine model suggest that some fluctuations in the GI simulator may have originated from insecure openings in the chamber, causing minor air leaks that affected pressure stability. The results suggest that the system’s control algorithms are reasonably well suited for managing gradual, biologically influenced changes in pressure within real tissue. However, further improvement in calibration and control algorithms is essential to better simulate and adapt to real-life physiological conditions, where living organs exhibit both passive and active responses through involuntary muscle contractions. Although the porcine model offers greater realism than the GI simulator does, it does not fully replicate the complex dynamics of living organs, such as active motility and rhythmic contractions, which introduce variability that demands more sophisticated control, especially for involuntary events such as coughing, belching, and spontaneous muscle contractions. Despite these challenges, the system effectively adapts to the porcine model, maintaining the desired pressure levels throughout the experiment. This adaptability underscores the system's potential for clinical applications, where variable tissue characteristics and dynamic physiological conditions are common. To enhance adaptability, AI-based techniques, such as machine learning or deep learning models, could be explored to fine-tune the control system, provided that sufficient data on these involuntary movements become available. Training the system with diverse datasets that capture a range of physiological responses would allow AI algorithms to dynamically adjust control parameters, anticipating and managing complex fluctuations. This approach could enhance the system’s ability to maintain target pressure despite unpredictable shifts in muscle tone or motility, providing robust adaptability in real-time clinical settings.

The compact design of the FPCB sensor module allows for integration into flexible endoscopes, including those used for small animals, and suggests that the system can also be incorporated into larger endoscopes used for humans or large animals. This integration enables simultaneous monitoring of pressure data and visual images, enhancing diagnostic accuracy and providing a comprehensive assessment of GI conditions. Unlike traditional methods that require protective belts, e.g., ColoWrap (ColoWrap, LLC, Durham, NC), to prevent rupture during procedures, the automatic pressure control system within the endoscope eliminates the need for such belts, offering a more comfortable, less restrictive experience and optimal abdominal space for inspection. This technology is particularly beneficial in veterinary care, where animals are fully anesthetized during procedures. Providing precise, real-time pressure control without external devices significantly improves the efficiency and effectiveness of veterinary diagnostics and treatment.

The potential applications of this system extend beyond endoscopic diagnostics. Its ability to provide reliable and precise pressure regulation makes it valuable in critical care settings, such as intensive care unit monitoring and surgical environments. Compared with similar studies that employed balloon-tipped catheters or fluid-filled methods for pressure measurement, our system showed greater consistency and stability in real-time pressure control [24]. Although it is generally assumed that the IAP can be measured consistently throughout the abdomen because it is a closed space filled with incompressible fluid, several studies have demonstrated a high correlation between intragastric pressure and the IAP [25–33]. This correlation suggests that the sensor module could be effectively utilized for IAP monitoring and management. The system offers distinct advantages over traditional IAP measurement methods by eliminating the risks associated with urinary infections, minimizing issues with over- or underdamping of pressure readings, reducing body position dependency, and providing continuous, noninvasive monitoring [24, 34]. Additionally, beyond GI endoscopy, this system could be adapted for use in other minimally invasive procedures where precise pressure control is critical, such as laparoscopy or thoracic surgeries. The findings from this study could contribute to the design of pressure management systems for other minimally invasive procedures. Additionally, installing a sensor near the end-effector (such as a surgical instrument) would allow for real-time pressure feedback precisely at the site of interest, enhancing procedural safety by preventing over- or under-insufflation. These principles could be applied to create more precise and adaptable systems in diverse clinical settings. To support broader applications, further research on system adaptability across various anatomical conditions, long-term efficacy studies in clinical trials, and comparisons of performance in different surgical environments are needed. Additionally, investigating how to optimize sensor placement near the end-effector for different tools and procedures is critical to improve accuracy and control. This would ensure that the system integrates well across diverse surgical tools and environments. With this integration, minimally invasive surgery could eliminate the need for an additional incision solely for pressure measurement.

While the study results are promising, several limitations must be considered. While the sensor module was tested in controlled environments, such as an acrylic chamber, an upper GI endoscopy simulator, and a porcine specimen, these conditions may not fully replicate the complexities of a living biological system. Physiological factors, such as tissue elasticity, peristalsis, GI motility (including irregular contractions), respiratory movements (such as breathing, coughing, and belching), natural gas production (through fermentation or air ingestion), and the presence of gastric fluids (covering or even submerging the sensor), could introduce variability in pressure readings [35]. These factors were not fully replicated in the simulator environment. Therefore, further testing in animal models is necessary to validate the sensor’s accuracy and reliability under more realistic physiological conditions. Additionally, since the system utilizes a suction channel for automatic control, it does not fully integrate with conventional endoscopic systems that require manual suction operations. For example, if the user intends to use suction for removing body fluids or blood, they might need to activate the air to increase the air inside the lumen artificially, thereby increasing the suction force. However, this issue could be resolved by electrifying the air, water, and suction functions, allowing the system to prioritize user-intended suction and override automatic controls when necessary. When next-generation electrified endoscopy systems become available, this system can be adopted immediately. Moreover, while the Bland‒Altman analysis indicates a high level of agreement between the sensor module and the reference gauge, the confidence intervals suggest that there could still be minor variations in some cases. These variations, although small, could become significant in critical care settings where even slight inaccuracies in pressure measurement could influence clinical decisions.

Future work should focus on refining the calibration process, fine-tuning the PID parameters, implementing hybrid fuzzy logic control, and leveraging AI-based feedback control algorithms, such as reinforcement learning, to further minimize discrepancies. The incorporation of machine learning or deep learning algorithms could enhance real-time sensor data processing, allowing the system to distinguish physiological noise from actual pressure variations. Additionally, adaptive filtering techniques may further refine the accuracy of sensor readings by dynamically adjusting to changing conditions, ensuring optimal sensor performance in critical care scenarios. These advancements could significantly increase the system’s suitability for clinical use, both in routine endoscopic procedures and critical care environments, where unexpected incidents can occur and be recognized with high accuracy. To support these improvements, we plan to collect data on involuntary movements, such as coughing, belching, and muscle contractions, during preclinical trials. These data provide essential insights for refining AI-based control algorithms and improving system responsiveness under realistic physiological conditions. Quantitative evaluations using key performance indicators, such as response times, standard errors, and time to steady-state, will measure the effectiveness of these improvements in both animal models and clinical settings. In future preclinical trials, subject selection will adhere to well-defined eligibility criteria to ensure the accuracy and generalizability of the results. For example, we plan to use beagles because of their frequent use in medical research because of their size and temperament, making them suitable for our study. The selection criteria will include factors such as age, health status, and the absence of pre-existing gastrointestinal conditions. This approach ensures comparability to other studies and generalizability to broader populations, improving patient safety, procedural efficiency, and pressure management during endoscopic and other minimally invasive procedures.

## Conclusion

Overall, this study demonstrated the feasibility and effectiveness of the sensor module and automated pressure control system for real-time monitoring and regulation of intragastric pressure during endoscopic procedures. By providing a reliable solution for maintaining optimal pressure, particularly in anesthetized patients, this system addresses a critical need for consistent pressure control, significantly enhancing both patient safety and procedural efficiency by minimizing risks associated with improper insufflation.

The system’s ability to maintain target pressure within an acceptable deviation was consistently demonstrated across all trials, where the results indicate its potential for broader applications beyond endoscopy, including in critical care and minimally invasive surgeries, where precise pressure management is essential. With further refinement, such as AI-driven algorithm enhancements based on clinical data, this system could become an invaluable tool for improving clinical outcomes across a range of medical procedures, providing a new standard for automated, real-time pressure control.

## Data Availability

The datasets generated and/or analyzed during the current study are available in the Science Data Bank repository, https://www.scidb.cn/en/anonymous/ejZKSnoy, upon filling out a Data Access Application form.
